# Supplementary material for: Reported practices related to, and capability to provide, first-line knee osteoarthritis treatments: a survey of 1064 Australian physical therapists
Source: Braz J Phys Ther. 2021 Sep 9;25(6):854–63. doi: 10.1016/j.bjpt.2021.08.001 (PMC8721054; doi:10.1016/j.bjpt.2021.08.001)
Supplement: Supplementary file 1 [file mmc1.docx]

**Checklist for Reporting Results of Internet E-Surveys (CHERRIES)**

**Reported practices related to, and capability to provide, first-line knee osteoarthritis treatments: A survey of 1064 Australian physiotherapists**

| ***Item*** | ***Checklist Item*** | ***Location*** |
| --- | --- | --- |
| **Design** | | |
|  | Survey design | Page 5-6 |
| **IRB (Institutional Review Board) approval and informed consent process** | | |
|  | IRB approval | Page 5 |
|  | Informed consent | Page 6 |
|  | Data protection | Page 6 |
| **Development and pre-testing** | | |
|  | Development and testing | Page 5-6 |
| **Recruitment process and description of the sample having access to the questionnaire** | | |
|  | Open survey vs. closed survey | Page 6 |
|  | Contact mode | Page 6 |
|  | Advertising the survey | Page 6 |
| **Survey administration** | | |
|  | Web/e-mail | Page 5-6 |
|  | Context | Page 6 |
|  | Mandatory / voluntary | Page 6 |
|  | Incentives | Page 6 |
|  | Randomization of items or questionnaires | Not applicable |
|  | Adaptive questioning | Supplementary file 1 |
|  | Number of items | Supplementary file 1 |
|  | Number of screens (pages) | Supplementary file 1 |
|  | Completeness check | Page 6 |
|  | Review step | Page 5-6 |
| **Response rates** | | |
|  | Unique site visitor | Not applicable |
|  | View rate (ratio of unique survey visitors / unique site visitors) | Not applicable |
|  | Participation rate (ratio of unique visitors who agreed to participate / unique first survey page visitors). | Page 7 |
|  | Completion rate (ratio of users who finished the survey/users who agreed to participate). | Page 7 |
| **Preventing multiple entries from the same individual** | | |
|  | Cookies / IP addresses used | Page 6 |
|  | Log file analysis | Not completed |
|  | Registration | Page 6 |
| **Analysis** | | |
|  | Handling of incomplete questionnaires | Page 6 |
|  | Questionnaires submitted with an atypical timestamp | Not addressed |
|  | Statistical correction | Not applicable |
