# Supplementary material for: Reported practices related to, and capability to provide, first-line knee osteoarthritis treatments: a survey of 1064 Australian physical therapists
Source: Braz J Phys Ther. 2021 Sep 9;25(6):854–63. doi: 10.1016/j.bjpt.2021.08.001 (PMC8721054; doi:10.1016/j.bjpt.2021.08.001)
Supplement: Supplementary file 2 [file mmc2.docx]

**Supplementary file 1** Physiotherapist survey

I willingly volunteer to participate in the study titled ‘Bridging the gap between evidence and practice in the management of chronic knee pain.’ I am happy to complete online questionnaires to explore my knowledge of current evidence and confidence in applying this evidence in the management of chronic knee pain. I am aware that I am free to withdraw participation at any time without penalty. Y/N

Throughout this questionnaire the following definitions related to confidence should be used:

**Very confident** – I have expert knowledge and skills on this area 
**Confident** – I know a lot on this area
**Average** – I know the basics on this area
**Below average** – This is an area where my knowledge can improve, 
**Not confident at all** – I know very little or nothing on this area

1. What setting do you work in?
   1. Public
   2. Private
   3. Both public and private
   4. Other (please specify)
2. What is your level of experience?
   1. <5 years as a qualified physiotherapist
   2. 5-10 years as a qualified physiotherapist
   3. 11-15 years as a qualified physiotherapist
   4. >15 years as a qualified physiotherapist
3. Have you completed any post-graduate training (Masters or PhD)? Y/N
4. Please rate your level of agreement with the following statements

(Strongly agree / Agree / Neither agree or disagree / Disagree / Strongly disagree)

- 1. I know how to deliver exercise and education to people with knee osteoarthritis following current clinical practice guidelines
  2. I have been trained in delivering exercise and education to people with knee osteoarthritis following current clinical practice guidelines
  3. I have the skills to deliver exercise and education to people with knee osteoarthritis following current clinical practice guidelines
  4. As a physiotherapist, it is my job to deliver exercise and education to people with knee osteoarthritis following current clinical practice guidelines
  5. I am confident I can deliver exercise and education to people with knee osteoarthritis following current clinical practice guidelines
  6. I am confident I can deliver exercise and education to people with knee osteoarthritis following current clinical practice guidelines, even when the patient is not motivated
  7. If I deliver exercise and education to people with knee osteoarthritis following current clinical practice guidelines, patient outcomes will be optimised
  8. If I deliver exercise and education to people with knee osteoarthritis following current clinical practice guidelines, patients will be more active
  9. In the organisation I work, all necessary resources are available to deliver exercise and education to people with knee osteoarthritis following current clinical practice guidelines

1. Please indicate the current level of evidence for the following active interventions for **knee osteoarthritis (Strongly supports / Supports / Unclear / Doesn’t support)**
   1. Strength exercise
   2. Aerobic exercise
   3. Neuromotor exercise
   4. Weight loss of 10% of body mass index
2. Please indicate your confidence in applying the following active interventions for **knee osteoarthritis (Very confident / Confident / Average / Below average / Not confident at all / I do not use this intervention)**
   1. Strength exercise
   2. Aerobic exercise
   3. Neuromotor exercise
3. Based on current evidence, please indicate if the following statements are true, false or unclear.
   1. Closed kinetic chain exercises are more effective than open kinetic chain exercises for **knee osteoarthritis**
   2. Supervised exercise programs are more effective than unsupervised exercises for **knee osteoarthritis**
   3. If a patient with **knee osteoarthritis** reports knee pain during an exercise, the exercise should always be stopped or changed
   4. There is no evidence to suggest that any particular exercise is more effective for **knee osteoarthritis**
   5. Physical inactivity is a risk factor for **knee osteoarthritis** progression
4. When treating people with knee osteoarthritis, how often do you

(All the time / Most of the time / Some of the time / Rarely / Never)

- 1. prescribe strength exercises
  2. prescribe aerobic exercise
  3. prescribe neuromotor exercise
  4. write down their exercise program
  5. provide video examples of their exercises
  6. write down other key education points
  7. refer them to additional online resources to help management
  8. discuss the possible importance weight loss to help manage their condition
  9. provide advice about physical activity levels
  10. refer for or provide supervised exercise programs
  11. refer for or provide home exercise programs

1. Please indicate your confidence in providing the following

(Very confident / Confident / Average / Below average / Not confident at all)

- 1. Patient education on physical activity participation (e.g. how much walking or running they should do)
  2. Patient education on contributing factors and the importance of self-management
  3. Discuss the importance of reducing or controlling weight to help manage their condition

1. Are you aware of any Clinical Practice Guidelines to guide the management of knee osteoarthritis? (Y / N)
2. Do you use any Clinical Practice Guidelines to guide the management of your knee osteoarthritis patients? (Y / N)
