# Supplementary material for: Reported practices related to, and capability to provide, first-line knee osteoarthritis treatments: a survey of 1064 Australian physical therapists
Source: Braz J Phys Ther. 2021 Sep 9;25(6):854–63. doi: 10.1016/j.bjpt.2021.08.001 (PMC8721054; doi:10.1016/j.bjpt.2021.08.001)
Supplement: Supplementary file 3 [file mmc3.docx]

**Reported practices related to, and capability to provide, first-line knee osteoarthritis treatments: A survey of 1064 Australian physical therapists**

**Running title: Practices and capability of Australian physical therapists treating osteoarthritis**

Barton CJ^1*^, Pazzinatto MF^1^, Crossley KM^1^, Dundules K^1^, Lannin NA^2^, Francis M^1^, Wallis J^3^, Kemp JL^1^

1 La Trobe Sport and Exercise Medicine Research Centre, School of Allied Health, Human Services and Sport, La Trobe University, Bundoora, Australia

2 Department of Neuroscience, Monash University, Melbourne, Australia; and Alfred Health, Melbourne, Australia

3 Monash Department of Clinical Epidemiology, Cabrini Institute, Australia

* Corresponding author: Dr Christian J. Barton; address: Health Sciences 3, La Trobe University, Bundoora, VIC 3068, Australia e-mail: [c.barton@latrobe.edu.au](mailto:c.barton@latrobe.edu.au)

**HIGHLIGHTS**

- Australian physical therapists typically prescribed strength-focused home exercise
- Aerobic and neuromuscular exercise are prescribed some of the time
- Confidence is associated with aerobic and neuromuscular exercise prescription frequency
- Less than half surveyed had been trained to deliver education and exercise therapy
- Just one in nine could name an accepted osteoarthritis guideline

**ABSTRACT**

**Background**

Physical therapists play a key role in providing first-line knee osteoarthritis treatments, including patient education and exercise therapy.

**Objectives**

Describe Australian physical therapists’ awareness of guidelines; reported practices; and beliefs about capability, opportunity, motivation, and evidence.

**Methods**

An online cross-sectional survey was completed by physical therapists prior to attending the Good Living with osteoArthritis from Denmark (GLA:D^®^) Australia training courses (March 2017 to December 2019). The survey instrument was developed by an expert panel and was informed by the Theoretical Domains Framework.

**Results**

1064 physical therapists from all Australian states and territories participated. 11% (n=121) could name an accepted guideline, 98% agreed it was their job to deliver patient education and exercise therapy, and 92% agreed this would optimise outcomes. Most reported providing strength exercise (99%), written exercise instructions (95%), treatment goal discussion (88%), and physical activity advice (83%) all or most of the time. Fewer provided aerobic exercise (66%), neuromuscular exercise (54%), and weight management discussion (56%) all or most of the time. Approximately one quarter (23-24%) believed they did not have the skills, knowledge, or confidence to provide education and exercise therapy recommended by guidelines, and just 48% agreed they had been trained to do so.

**Conclusion:** Australian physical therapist treating knee osteoarthritis typically provide strength-based home exercise with written instructions, alongside goal setting and physical activity advice. Just one in nine could name a guideline. Education and training activities are needed to support physical therapists to access, read and implement guidelines, especially for aerobic and neuromuscular exercise, and weight management.

**Keywords:** education; evidence; exercise; osteoarthritis; Physical therapy.

**INTRODUCTION**

Clinical practice guidelines consistently recommend patient education, exercise therapy, and weight management as first-line care for people with osteoarthritis.^1-4^ Patient education combined with exercise therapy has compelling evidence of effectiveness^5^ and cost-effectiveness,^6,7^ and can avert the need for surgery,^8,9^ potentially providing substantial health system savings.^10^ Alongside condition specific knowledge and treatment options, key recommended foci for patient education for people with knee osteoarthritis include promoting the importance of self-management, goal setting, exercise and physical activity, and weight management.^2,4,11^ There appears to be no one form of exercise therapy with superior outcomes,^5^ with guidelines recommending varying combinations of strength, aerobic, and neuromuscular exercise.^1-4^

Physical therapists play a key role in implementing patient education and exercise therapies to people with knee osteoarthritis.^12^ Therefore, they must possess requisite capability, opportunity, and motivation to do so.^13-15^ These key enablers of guideline implementation in primary care^16^ are currently not well understood in regard to physical therapists’ provision of first-line care to people with knee osteoarthritis. Available research indicates positive attitudes toward evidence-based practice^17^ and a belief among most physical therapists that patient education, exercise therapy, and weight management are key treatments for people with knee osteoarthritis.^18^ A recent mixed-methods study of Australian-based physical therapists working in a single hospital outpatient setting reported high confidence in strength exercise prescription, but not in aerobic exercise prescription, or pain and weight management.^19^ These likely skills gaps related to pain and weight management have been further highlighted in a recent large survey based study including physical therapists, physical therapy students, nurses, general practitioners, and registrars from Australia, New Zealand, and Canada.^20^ A deeper understanding of physical therapy practice related to first-line care for knee osteoarthritis, along with capability, opportunity, and motivation to implement, may help guide professional development initiatives in Australia.

The primary aims of this study were to describe Australian physical therapists’ (i) awareness of guidelines; (ii) reported practices related to first-line treatments; (iii) beliefs about capability, opportunity, and motivation to provide first-line treatments; (iv) beliefs about evidence supporting, and confidence to implement, specific exercise prescription and weight management discussion; and (v) beliefs regarding exercise and physical activity practices. Secondary aims of this study were to determine the relationship of reported exercise prescription and weight management discussion with beliefs about evidence supporting these practices, confidence to implement these practices, post-graduate training completion and clinic setting; and to provide proposed evidence-based behavior change intervention functions^21^ to address evidence-practice gaps identified.

**METHODS**

The research design was a cross-sectional survey of physical therapists. Reporting of this study has been guided by the Checklist for Reporting Results of Internet E-Surveys (CHERRIES).^21^ Ethical approval was granted by La Trobe University’s Human Ethics Research Committee (S16-51).

**Development and testing**

The online survey (Supplemental Online Material) was developed by members of the research team (CJB, NAL, KMC, JLK), and guided by osteoarthritis guidelines.^1-4^ Questions covered (i) awareness (yes or no) and ability to name accepted knee osteoarthritis guidelines; (ii) beliefs about physical and psychological capabilities, physical and social opportunity, and reflective motivation to provide education and exercise therapy to people with knee osteoarthritis; (iii) reported practices related to education and exercise therapy; (iv) beliefs about evidence supporting, and confidence to implement, specific exercise prescription and weight management support; and (v) beliefs regarding exercise and physical activity practices when treating people with knee osteoarthritis. Questions related to beliefs about capability, opportunity, and motivation were structured based on components of the Theoretical Domains Framework.^13,15^ Participant characteristics including years of experience and post-graduate training completion were also collected.

The survey was piloted by members of the research team and three physical therapists working in the lead author’s (XX) clinical network. The final survey was administered using ‘Survey Monkey®’ (San Mateo, United States) software. The opening page included an information statement outlining the expected length of time to complete the survey (12 minutes), how data would be stored and protected, who the chief investigator was, the purpose of the study and a tick box for informed consent.

**Recruitment**

Data were collected on a convenience sample of physical therapists attending a Good Living with osteoArthritis from Denmark (GLA:D^®^) Australia training courses between March 2017 and December 2019. Participation occurred during the first 15 minutes of these training courses, before any content was taught, as a standard component of the training. Training course attendees who did not wish to participate could either not open the survey link shared, or decline consent on the first page, which would end the survey.

**Data synthesis and analysis**

Primary and secondary aims of the study (see Supplementary file 1.2) were exported from ‘Survey Monkey^®^’ to Excel for synthesis and analysis. All data were analysed anonymously.

*Primary aims:* The proportion of physical therapists stating they were aware of, and able to name an accepted guideline in free text was evaluated. To be considered an ‘accepted’ guideline for knee osteoarthritis, the named guideline needed to focus on osteoarthritis and be from a recognised professional body, e.g. Osteoarthritis Research Society International (OARSI).^2,4^

Reported practices related to education and exercise therapy when treating people with knee osteoarthritis were summarised as the proportion who stated ‘never’, ‘rarely’, ‘some of the time’, ‘most of the time’, and ‘all the time’. Beliefs about capability, opportunity and motivation were summarised as the proportion who stated ‘strongly agree’, ‘agree’, ‘neither agree or disagree’, ‘disagree’, and ‘strongly disagree’. Beliefs about evidence supporting specific forms of exercise prescription (strength, aerobic, neuromuscular) and weight management were summarised as the proportion who stated ‘strongly supports’, ‘supports’, ‘unclear’, and ‘does not support’. Confidence to implement specific forms of exercise (strength, aerobic, neuromuscular) and weight management were summarised as the proportion who stated ‘very confident’, ‘confident’, ‘average’, ‘below average’, ‘not confident at all’, and ‘I do not use this intervention’. Beliefs about exercise and physical activity practices were summarised as the proportion who stated ‘true’, ‘false’, and ‘unclear’.

*Secondary aims:* Chi-square tests for independence (*X*^2^) were used to determine the relationship between practices (all or most of the time) of specific exercise prescription (strength, aerobic, neuromuscular), and weight management with (i) beliefs about evidence supporting (supports/strongly supports) and confidence to implement (confident/very confident) these specific interventions, (ii) post-graduate training completion (yes/no), and (iii) clinic setting (public/private). Effect sizes (ES) were calculated using Phi for 2x2 contingency table and Cramer’s V for tables larger than 2x2 contingency table. Chi-square comparisons were completed in Statistical Package for the Social Sciences (SPSS) version 26 with an α level set at 0.05. Effect sizes were categorised as negligible (< 0.10), small (≥ 0.10), moderate (≥ 0.30) or large (≥ 0.50).^22^

Only relationships categorised as small or greater were considered relevant to inform recommendations to address evidence-practice gaps. For a finding to be considered an important evidence-practice gap needing to be addressed, at least 10% of respondents were required to: (i) not provide the guideline-recommended intervention all or most of the time; (ii) not agree or strongly agree with the statement; (iii) not to report confidence in providing the intervention; or (iv) not to believe the intervention had supporting evidence.

**RESULTS**

1064 respondents (out of 1068) of varying years of experience (362 [34%] <5 years; 208 [20%] 5-10 years; 113 [11%] 11-15 years; 381 [36%] >15 years) consented to participate. The remaining four participants did not start the survey. 379 (37%) had post-graduate training (Masters or PhD); and 746 (73%) were from private settings, 205 (20%) were from public settings, and 77 (7%) were from mixed private and public settings.

*Primary outcomes:* Thirty-eight percent (401/1064) of respondents stated they were aware of clinical practice guidelines for the management of knee osteoarthritis, with 11% (121/1064) being able to name at least one accepted guideline. The most commonly named guidelines were OARSI^2 4^ (4%, n=47), National Institute for Health Care Excellence (NICE)^23^ (4%, n=46), and Royal Australian College of General Practitioner (RACGP)^1^ (4%, n=43). Reported education and exercise therapy practices are presented in **Figure 1**. Beliefs about capability, opportunity, and motivation to implement education and exercise therapy following current clinical practice guidelines are presented in **Figure 2**. Reported confidence to implement specific exercise prescription and weight management education practices are presented in **Figure 3**. Beliefs about evidence supporting specific exercise prescription and weight loss; and exercise prescription and physical activity are presented in **Figure 4**.

*Secondary outcomes:* The relationships between providing specific exercise prescription and discussing weight management all or most of the time with beliefs about evidence and confidence to provide these interventions, completion of post-graduate training, and clinic setting are presented in **Table 1**. Proposed evidence-based behaviour change intervention functions mapped to key survey findings are summarised in **Table 2**.

**Table 1** The relationship (*X*^2^ tests for independence) between providing specific exercise (strength, aerobic, neuromuscular) prescription and discussing weight management all or most of the time with beliefs about evidence and confidence to provide the intervention, completion of post-graduate training, and clinic setting.

|  | Confident or very confident to provide | | Believe evidence supports or strongly supports | | Have completed post-graduate training | | Clinic setting  (public/private) | |
| --- | --- | --- | --- | --- | --- | --- | --- | --- |
| Practice  (provide all of most of the time) | p-value | Effect size | p-value | Effect size | p-value | Effect size | p-value | Effect size |
| Strength exercise | 0.848 | 0.01 | 0.652 | 0.01 | 0.585 | 0.02 | 0.881 | 0.01 |
| Aerobic exercise | **<0.001** | **0.29** | **<0.001** | **0.29** | 0.104 | 0.04 | 0.645 | 0.02 |
| Neuromuscular exercise | **<0.001** | **0.46** | **<0.001** | **0.27** | **0.001** | **0.12** | **0.028** | **0.07^#^** |
| Discuss weight management | **<0.001** | **0.46** | **0.001** | **0.11** | **<0.001** | **0.13** | **<0.001** | **0.12^** |

# More likely in private setting; ^ More likely in public setting

**Bolded text =** statistically significant relationship; effect sizes categorised as negligible (< 0.10), small (≥ 0.10), moderate (≥ 0.30) or large (≥ 0.50).^22^

**Table 2** Survey findings related to delivering guideline-recommended education and exercise therapy mapped to proposed evidence-based behaviour change intervention functions.^24^

| **COM-B** | **TDF** | **Key survey findings* related to delivering guideline-recommended education and exercise therapy** | **Proposed evidence-based behaviour change intervention functions** |
| --- | --- | --- | --- |
| Capability -physical | Training and skills | 23% did not agree they had the skills.  52% did not agree they had been trained.  *Small relationship* between post-graduate training completion and providing neuromuscular exercise and discussing weight management all or most of the time. | ***Training*** in how to provide patient education and exercise therapy, particularly in those without post-graduate training. |
| Capability-psychological | Knowledge | 24% of respondents did not agree they knew how.  20% and 13% did not believe evidence supported aerobic and neuromuscular exercise.  *Knowledge and practice (all or most of the time):* small relationship between believing evidence supported and aerobic and neuromuscular exercise and discussing weight management. | ***Education and training*** about how to provide patient education and exercise therapy.  ***Education*** about evidence supporting aerobic and neuromuscular exercise. |
| Opportunity-physical | Organisational resources | 34% did not agree their organisation had all the necessary resources  *Small relationship* working in a public setting and discussing weight-management all or most of the time. | ***Environmental restructuring and enablement*** to ensure appropriate resources (time and facilities) and support to deliver exercise therapy, and dietetics access (e.g. reflecting public settings). |
| Motivation-  Reflective | Beliefs about capabilities | 24% were not confident, which increased to 53% when the patient was not motivated.  *Specific education:* 30%, 39% and 49% were not confident providing education related to self-management, physical activity participation and weight management respectively.  *Specific exercise:* 29%, 46% and 62% were not confident providing strength, aerobic and neuromuscular exercise respectively  *Confidence and practice (all or most of the time):* moderate relationship between greater confidence and providing neuromuscular exercise and discussing weight management; small relationship between greater confidence providing aerobic exercise. | ***Education*** to provide guidance on how to provide patient education (focused on self-management, physical activity and weight management) and all forms of exercise therapy (particularly neuromuscular and aerobic exercise), with an emphasis on unmotivated patients.  ***Persuasion and incentivisation*** to facilitate more frequent aerobic and neuromuscular exercise, and weight management discussion. |

* Findings where intervention was indicated for ≥ 10% of respondents; or where there was a relationship of small (effect size ≥ 0.10) or greater size.

COM-B = Capability, Opportunity, Motivation – Behaviour domains; TDF = Theoretical Domains Framework domains.

**DISCUSSION**

Most Australian physical therapists believe it is their job to deliver guideline-recommended care, and that doing so will optimise patient outcomes, yet struggled to name an accepted guideline. Aligning with guideline-recommended care,^1-4^ most respondents (83-88%) reported patient education practices including facilitating goal setting and providing physical activity advice all or most of the time, which can optimise exercise adherence and self-management.^25,26^ Additionally, 99% of respondents reported providing strength exercise all or most of the time, which is a guideline recommended exercise therapy^1-4^ capable of improving pain and function,^5^ and reducing the risk of osteoarthritis progression.^27^

Our findings indicate that strength exercise prescription is embedded in Australian physical therapy practice for knee osteoarthritis, regardless of confidence to provide it, post-graduate training completion or clinical setting (public/private). Aerobic and neuromuscular exercise were prescribed less often than strength, despite equivalent evidence supporting these exercise therapies to improve pain and function in people with knee osteoarthritis.^5^ Aerobic exercise has the added benefit of improving comorbidity management,^28^ and neuromuscular exercise can assist in allowing a substantial proportion of people with knee osteoarthritis to delay and potentially avoid surgery.^8,9^

Exercise therapy appears to be primarily prescribed by Australian physical therapists via home-based programs, guided by written instructions to enhance adherence. Just one in four provided video instructions, despite the perceived value of exercise videos among patients, and their potential to improve adherence.^29^ Low use of video instructions may reflect preferences of people with knee osteoarthritis or inadequate time of physical therapists during consultation. Compared to home-based exercise programs, far fewer respondents reported facilitating supervised exercise programs to all or most of the time, despite nine out of ten respondents believing supervised exercise was more effective. The discord between implementation and perceived value of supervised exercise may be explained by limited organisational resources identified in this study, and other supervised exercise participation barriers identified in previous research, including conflicting demands on patient time (family, work) and out of pocket costs associated with supervision.^30^

Approximately half of our respondents reported writing down key education points, and just 12% referred to additional online resources all or most of the time. Given that recall of verbally provided medical information can be 20% or lower,^31,32^ bridging this evidence-practice gap and supporting physical therapists to provide additional education resources to people with knee osteoarthritis is encouraged. Recent cross-sectional work indicates the provision of printed information by physical therapists alongside prescription of self-management strategies, makes it three times more likely these strategies will be adhered to.^33^ Since internet content related to musculoskeletal pain education is often inaccurate, including on non-commercial websites, development and evaluation of accurate and trusted online resources for people with knee osteoarthritis should be a research priority.^34,35^

We identified a large evidence-practice gap related to weight-management in Australian physical therapists. Ninety-six percent of respondents believed weight loss was supported by evidence for knee osteoarthritis, but only 56% discussed weight management all or most of the time. Additionally, one in two physical therapists in this study were not confident to discuss weight management, a finding which is consistent with low confidence reported in qualitative research with physical therapists working in Australia.^19^ Addressing this evidence-practice gap is especially important, considering most people with knee osteoarthritis presenting to physical therapists are over-weight,^36^ and they appear open to physical therapists providing weight management support to them if they have adequate knowledge and skills.^37^

***Recommendations to bridge current evidence-practice gaps***

Our findings highlight numerous barriers to physical therapists providing guideline-recommended education and exercise therapy for knee osteoarthritis, allow us to propose numerous potential solutions (Table 2). Better guideline dissemination may help, with just one in nine respondents able to name an accepted knee osteoarthritis clinical practice guideline. This indicates most either did not consult guidelines or could not recall guidelines they consulted. However, considering criticism of osteoarthritis guidelines for a lack of guidance on how to provide recommended care,^3,38^ we encourage a far broader consideration of potential strategies to bridge the evidence-practice gaps we have identified. We strongly encourage education and training of Australian physical therapists, with nearly a quarter of respondents feeling they did not have the skills or knowledge to deliver, and less than half agreed they had been trained to deliver, guideline-recommended education and exercise therapy to people with knee osteoarthritis.^13,24^ This recommendation is further emphasised by the identified relationships between believing there is supporting evidence for, and frequency of providing, aerobic and neuromuscular exercise and education about weight management.

Education initiatives to improve physical therapist confidence is strongly encouraged, with findings indicating one in four were not confident to provide education and exercise therapy to people with knee osteoarthritis.^24^ Additionally, more than half were not confident in managing unmotivated patients, and a large proportion (29-49%) reported average or lower confidence to deliver the specific components of education and exercise therapy we surveyed. This included interventions like strength exercise and physical activity participation education, which most provided all or most of the time, indicating a substantial proportion of physical therapists frequently provide interventions they are not confident with. Our secondary analysis indicates that addressing confidence through education may be important to increase the frequency of aerobic and neuromuscular exercise prescription, and weight management discussion, from Australian physical therapists when treating people with knee osteoarthritis. Additional behaviour change techniques warranting consideration to increase the frequency in which these guideline recommended interventions are provided, include persuasion (e.g. marketing, patient stories) and incentivisation.^24^ Physicaltherapist training and support to implement guideline recommended care is increasingly being offered (e.g. GLA:D^®^ Australia,^39^ NSW Osteoarthritis Chronic Care Program^40^) and taken up by health services in Australia.^41^ However, key barriers to uptake of edcuation and exercise therapy by people with osteoarthritis identified in Australia^30,42,43^ include health system funding, and referrer (e.g. general practioner) buy in. Therefore, incentivisation targetting both program delivery (e.g. improved service funding) and program referral (e.g. payments for doctors to refer) could be considered.

Approximately one third of physical therapists did not believe their organisation had the necessary resources to provide guideline-recommended education and exercise therapy. This highlights a potential need for environmental restructuring and enablement to increase time available to deliver care, and provide facilities for physical therapists to deliver exercise therapy (e.g. dedicated exercise space and equipment).^24^ Further, improved funding for physical therapy services, and implementation of novel service delivery models to improve efficiency (e.g. group supervision) and access (e.g. telehealth) should also be considered to better enable the implementation of education exercise therapy for people with knee osteoarthritis.^24,30^

***Limitations***

The generalisability of our sample requires consideration. Physical therapists were recruited from GLA:D^®^ Australia training, which provides training to implement education and exercise therapy for osteoarthritis. Therefore, applicability of results may be limited to Australian physical therapy practice. Further work repeating our evaluation in physical therapist samples from other countries is encouraged. Participants were likely to be aware of their limitations and motivated to learn, and potentially less confident than those not choosing to attend training. Additionally, though, they may have had some understanding about the importance of first-line care for knee osteoarthritis. However, our response rate was greater than 99% and our cohort represented an estimated 4% of Australian physical therapists in all states and territories (1,064/~26,000^44^). It is not clear how accurately participants’ survey responses may reflect their actual clinical practice. Nonetheless, responses were anonymous, improving the likelihood of honest responses. Our survey included a limited number of questions, and did not cover all potential barriers and enablers to physical therapists providing guideline-recommended care to people with knee osteoarthritis.^13,24^ Future qualitative research (e.g. focus groups) is encouraged to explore findings of this study in more detail, including how to address identified guideline-practice gaps. When considering the broader socioecological context,^45^ other potential practice influences should also be considered, including at community (e.g. culture, patient expectations) and policy (e.g. funding) levels.^16,33^

**Conclusion**

Most aspects of Australian physical therapy practice align with guideline-recommended care, including the provision of education related to goal setting and physical activity, and strength-based home exercise programs. Yet, few physical therapists could name an accepted guideline, indicating limited direct engagement with these information sources. Nearly a quarter felt they did not have the skills, knowledge or confidence to deliver, and less than half agreed they had been trained to deliver, guideline-recommended education and exercise therapy. Education and training activities are needed to support physical therapists to access, read and implement guidelines are needed, especially for aerobic and neuromuscular exercise, and weight management.

**Acknowledgements:** CJB led this work as part of a a MRFF Translating Research Into Practice (TRIP) Fellowship (GNT1150439).

**Conflict of interests:** CJB is supported by a MRFF Translating Research Into Practice (TRIP) Fellowship (GNT1150439). JLK is supported by an NHMRC early career fellowship (GNT1119971). NAL is supported by a Future Leader Fellowship (GNT102055) from the National Heart Foundation of Australia. CJB, KMC and JLK lead the ‘not-for-profit’ implementation initiative, GLA:D® Australia, which trains Australian physical therapists to implement guideline-recommended education and exercise therapy.

**References**

1. RACGP. Guideline for the management of hip and knee osteoarthritis. Melbourne, 2018.

2. Bannuru RR, Osani MC, Vaysbrot EE, et al. OARSI guidelines for the non-surgical management of knee, hip, and polyarticular osteoarthritis. *Osteoarthritis Cartilage* 2019;27(11):1578-89. doi: 10.1016/j.joca.2019.06.011 [published Online First: 2019/07/03]

3. Nelson AE, Allen KD, Golightly YM, et al. A systematic review of recommendations and guidelines for the management of osteoarthritis: The chronic osteoarthritis management initiative of the U.S. bone and joint initiative. *Semin Arthritis Rheum* 2014;43(6):701-12. doi: 10.1016/j.semarthrit.2013.11.012 [published Online First: 2013/12/04]

4. McAlindon TE, Bannuru RR, Sullivan MC, et al. OARSI guidelines for the non-surgical management of knee osteoarthritis. *Osteoarthritis Cartilage* 2014;22(3):363-88. doi: 10.1016/j.joca.2014.01.003

5. Juhl C, Christensen R, Roos EM, et al. Impact of exercise type and dose on pain and disability in knee osteoarthritis: a systematic review and meta-regression analysis of randomized controlled trials. *Arthritis Rheumatol* 2014;66(3):622-36. doi: 10.1002/art.38290

6. Abbott JH, Wilson R, Pinto D, et al. Incremental clinical effectiveness and cost effectiveness of providing supervised physiotherapy in addition to usual medical care in patients with osteoarthritis of the hip or knee: 2-year results of the MOA randomised controlled trial. *Osteoarthritis Cartilage* 2018;27(3):424-434. doi: 10.1016/j.joca.2018.12.004

7. Skou ST, Roos EM, Laursen M, et al. Cost-effectiveness of 12 weeks of supervised treatment compared to written advice in patients with knee osteoarthritis: a secondary analysis of the 2-year outcome from a randomized trial. *Osteoarthritis Cartilage* 2020;28(7):907-916. doi: 10.1016/j.joca.2020.03.009

8. Skou ST, Roos EM, Laursen MB, et al. A Randomized, Controlled Trial of Total Knee Replacement. *N Engl J Med* 2015;373(17):1597-606. doi: 10.1056/NEJMoa1505467

9. Skou ST, Roos EM, Laursen MB, et al. Total knee replacement and non-surgical treatment of knee osteoarthritis: 2-year outcome from two parallel randomized controlled trials. *Osteoarthritis Cartilage* 2018;26(9):1170-1180. doi: 10.1016/j.joca.2018.04.014

10. Ackerman I, Skou S, Roos E, et al. Implementing a national first-line management program for moderate-severe knee osteoarthritis in Australia: A budget impact analysis focusing on knee replacement avoidance. *Osteoarthritis and Cartilage Open* 2020;2(3) 100070. doi: [10.1016/j.ocarto.2020.100070](https://doi.org/10.1016/j.ocarto.2020.100070)

11. French SD, Bennell KL, Nicolson PJ, et al. What do people with knee or hip osteoarthritis need to know? An international consensus list of essential statements for osteoarthritis. *Arthritis Care Res (Hoboken)* 2015;67(6):809-16. doi: 10.1002/acr.22518

12. Briggs A, Hinman R, Darlow B, et al. Confidence and attitudes toward osteoarthritis care among the current and emerging health workforce: A multinational interprofessional study. *American College of Rheumatology* 2019;1(4):219-35.

13. Huijg JM, Gebhardt WA, Dusseldorp E, et al. Measuring determinants of implementation behavior: psychometric properties of a questionnaire based on the theoretical domains framework. *Implement Sci* 2014;9:33. doi: 10.1186/1748-5908-9-33

14. Michie S, Johnston M, Abraham C, et al. Making psychological theory useful for implementing evidence based practice: a consensus approach. *Qual Saf Health Care* 2005;14(1):26-33. doi: 10.1136/qshc.2004.011155

15. Lawton R, Heyhoe J, Louch G, et al. Using the Theoretical Domains Framework (TDF) to understand adherence to multiple evidence-based indicators in primary care: a qualitative study. *Implement Sci* 2016;11:113. doi: 10.1186/s13012-016-0479-2

16. Rubio-Valera M, Pons-Vigués M, Martínez-Andrés M, et al. Barriers and facilitators for the implementation of primary prevention and health promotion activities in primary care: a synthesis through meta-ethnography. *PLoS One* 2014;9(2):e89554. doi: 10.1371/journal.pone.0089554

17. Condon C, McGrane N, Mockler D, et al. Ability of physiotherapists to undertake evidence-based practice steps: a scoping review. *Physiotherapy* 2016;102(1):10-9. doi: 10.1016/j.physio.2015.06.003

18. Teo PL, Hinman RS, Egerton T, et al. Identifying and Prioritizing Clinical Guideline Recommendations Most Relevant to Physical Therapy Practice for Hip and/or Knee Osteoarthritis. *J Orthop Sports Phys Ther* 2019;49(7):501-12. doi: 10.2519/jospt.2019.8676

19. Tang CY, Pile R, Croft A, et al. Exploring Physical Therapist Adherence to Clinical Guidelines When Treating Patients With Knee Osteoarthritis in Australia: A Mixed Methods Study. *Phys Ther* 2020;100(7):1084-1093. doi: 10.1093/ptj/pzaa049

20. Briggs AM, Houlding E, Hinman RS, et al. Health professionals and students encounter multi-level barriers to implementing high-value osteoarthritis care: a multi-national study. *Osteoarthritis Cartilage* 2019;27(5):788-804. doi: 10.1016/j.joca.2018.12.024

21. Eysenbach G. Improving the quality of Web surveys: the Checklist for Reporting Results of Internet E-Surveys (CHERRIES). *J Med Internet Res* 2004;6(3):e34. doi: 10.2196/jmir.6.3.e34

22. Cohen J. A power primer. *Psychol Bull* 1992;112(1):155-9.

23. NICE. Osteoarthritis: care and management, 2014.

24. Michie S, van Stralen MM, West R. The behaviour change wheel: a new method for characterising and designing behaviour change interventions. *Implement Sci* 2011;6:42. doi: 10.1186/1748-5908-6-42

25. Meade LB, Bearne LM, Sweeney LH, et al. Behaviour change techniques associated with adherence to prescribed exercise in patients with persistent musculoskeletal pain: Systematic review. *Br J Health Psychol* 2019;24(1):10-30. doi: 10.1111/bjhp.12324

26. Room J, Hannink E, Dawes H, et al. What interventions are used to improve exercise adherence in older people and what behavioural techniques are they based on? A systematic review. *BMJ Open* 2017;7(12):e019221. doi: 10.1136/bmjopen-2017-019221

27. Øiestad BE, Juhl CB, Eitzen I, et al. Knee extensor muscle weakness is a risk factor for development of knee osteoarthritis. A systematic review and meta-analysis. *Osteoarthritis Cartilage* 2015;23(2):171-7. doi: 10.1016/j.joca.2014.10.008

28. Skou ST, Pedersen BK, Abbott JH, et al. Physical Activity and Exercise Therapy Benefit More Than Just Symptoms and Impairments in People With Hip and Knee Osteoarthritis. *J Orthop Sports Phys Ther* 2018;48(6):439-47. doi: 10.2519/jospt.2018.7877

29. Ouegnin A, Valdes K. Client preferences and perceptions regarding a written home exercise program or video self-modeling: A cross-sectional study. *J Hand Ther* 2020;33(1):67-72. doi: 10.1016/j.jht.2018.09.006

30. Wallis J, Ackerman I, Brusco N, et al. Barriers and enablers to uptake of a contemporary guideline-based management program for hip and knee osteoarthritis: a qualitative study. *Osteoarthritis and Cartilage Open* 2020; 2(4):100095. doi**:** [10.1016/j.ocarto.2020.100095](https://doi.org/10.1016/j.ocarto.2020.100095)

31. Sherlock A. Response to Re: Patients' recollection and understanding of informed consent: a literature review. *ANZ J Surg* 2014;84(9):695. doi: 10.1111/ans.12757

32. Kessels RP. Patients' memory for medical information. *J R Soc Med* 2003;96(5):219-22. doi: 10.1258/jrsm.96.5.219

33. Peek K, Carey M, Mackenzie L, et al. Characteristics associated with high levels of patient-reported adherence to self-management strategies prescribed by physiotherapists. *International Journal of Therapy and Rehabilitation* 2020;27(1):1759-79.

34. de Oliveira Silva D, Rathleff MS, Holden S, et al. Patients and clinicians managing patellofemoral pain should not rely on general web-based information. *Phys Ther Sport* 2020;45:176-80. doi: 10.1016/j.ptsp.2020.07.004

35. Ferreira G, Traeger AC, Machado G, et al. Credibility, Accuracy, and Comprehensiveness of Internet-Based Information About Low Back Pain: A Systematic Review. *J Med Internet Res* 2019;21(5):e13357. doi: 10.2196/13357

36. Skou ST, Roos EM. Good Life with osteoArthritis in Denmark (GLA:D™): evidence-based education and supervised neuromuscular exercise delivered by certified physiotherapists nationwide. *BMC Musculoskelet Disord* 2017;18(1):72. doi: 10.1186/s12891-017-1439-y

37. Allison K, Delany C, Setchell J, et al. A qualitative study exploring the views of individuals with knee osteoarthritis on the role of physiotherapists in weight management: A complex issue requiring a sophisticated skill set. *Musculoskeletal Care* 2019;17(2):206-14. doi: 10.1002/msc.1391

38. Lin I, Wiles LK, Waller R, et al. Poor overall quality of clinical practice guidelines for musculoskeletal pain: a systematic review. *Br J Sports Med* 2018;52(5):337-43. doi: 10.1136/bjsports-2017-098375

39. Roos EM, Barton CJ, Davis AM, et al. GLA:D to have a high-value option for patients with knee and hip arthritis across four continents: Good Life with osteoArthritis from Denmark. *Br J Sports Med* 2018;52:1544-1545. doi: 10.1136/bjsports-2017-098904

40. Eyles JP, Bowden JL, Redman S, et al. Barriers and enablers to the implementation of the Australian Osteoarthritis Chronic Care Program (OACCP). *Osteoarthritis and Cartilage* 2020;28:S446.

41. Barton C, J K, Roos E, et al. Program Evaluation of GLA:D(R) Australia: Physiotherapist training outcomes and effectiveness of implementation for people with knee osteoarthritis. *Osteoarthritis and Cartilage Open* 2021;In Press doi: 10.21203/rs.3.rs-169093/v1

42. Barton CJ, Ezzat AM, Bell EC, et al. Knowledge, confidence and learning needs of physiotherapists treating persistent knee pain in Australia and Canada: A mixed-methods study. *Physiotherapy Theory and Practice* 2021;In Press

43. Barton CJ, King MG, Dascombe B, et al. Many physiotherapists lack preparedness to prescribe physical activity and exercise to people with musculoskeletal pain: A multi-national survey. *Phys Ther Sport* 2021;49:98-105. doi: 10.1016/j.ptsp.2021.02.002

44. WCPT. World Physiotherapy: World Confederation for Physical Therapy; 2020 [Available from: <https://world.physio/> accessed July 28th 2020.

45. McLeroy KR, Bibeau D, Steckler A, et al. An ecological perspective on health promotion programs. *Health Educ Q* 1988;15(4):351-77. doi: 10.1177/109019818801500401

**Figure and Table captions**

**
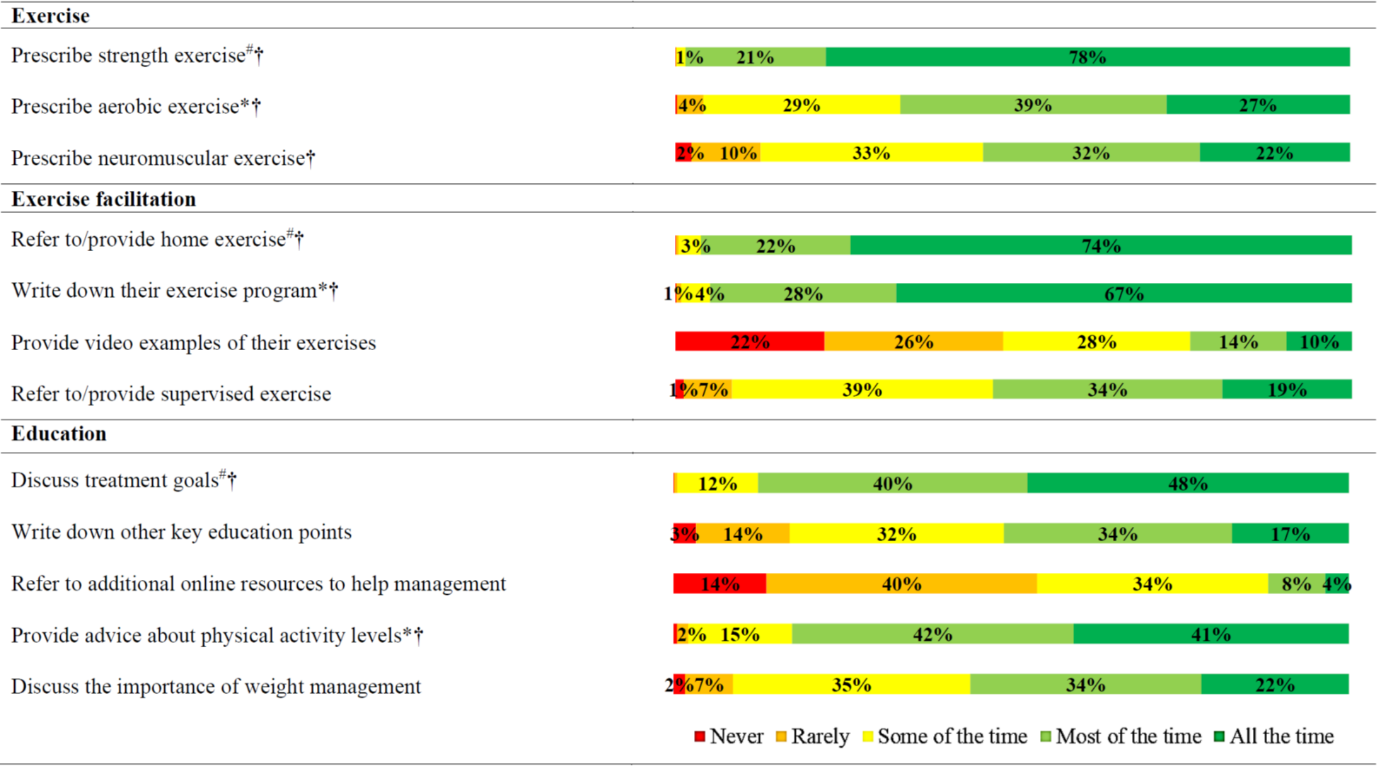
**

**Figure 1** Reported education and exercise therapy practices when treating people with knee osteoarthritis.

**
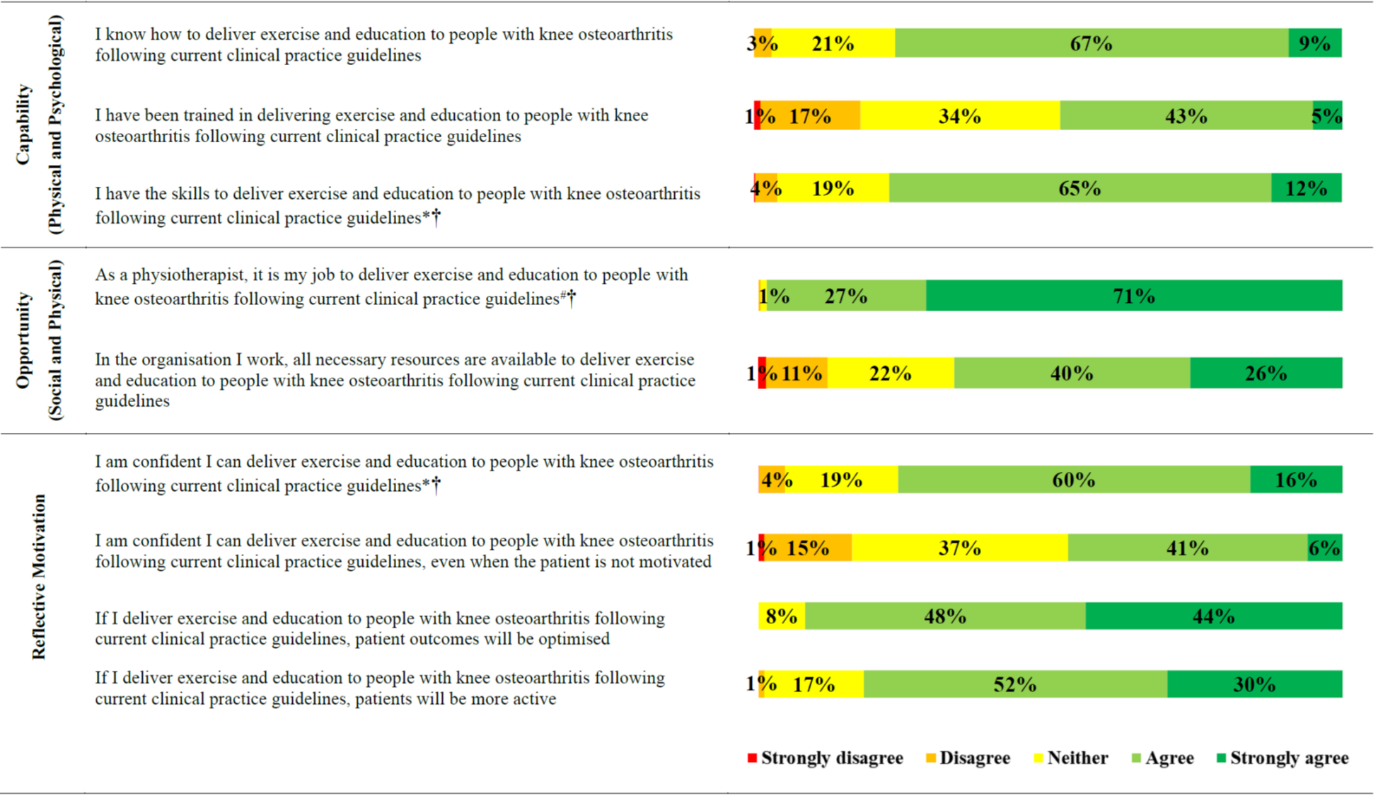
**

**Figure 2** Beliefs about capabilities, opportunities and motivations related to implementing education and exercise therapy people with knee osteoarthritis following current clinical practice guidelines.

**
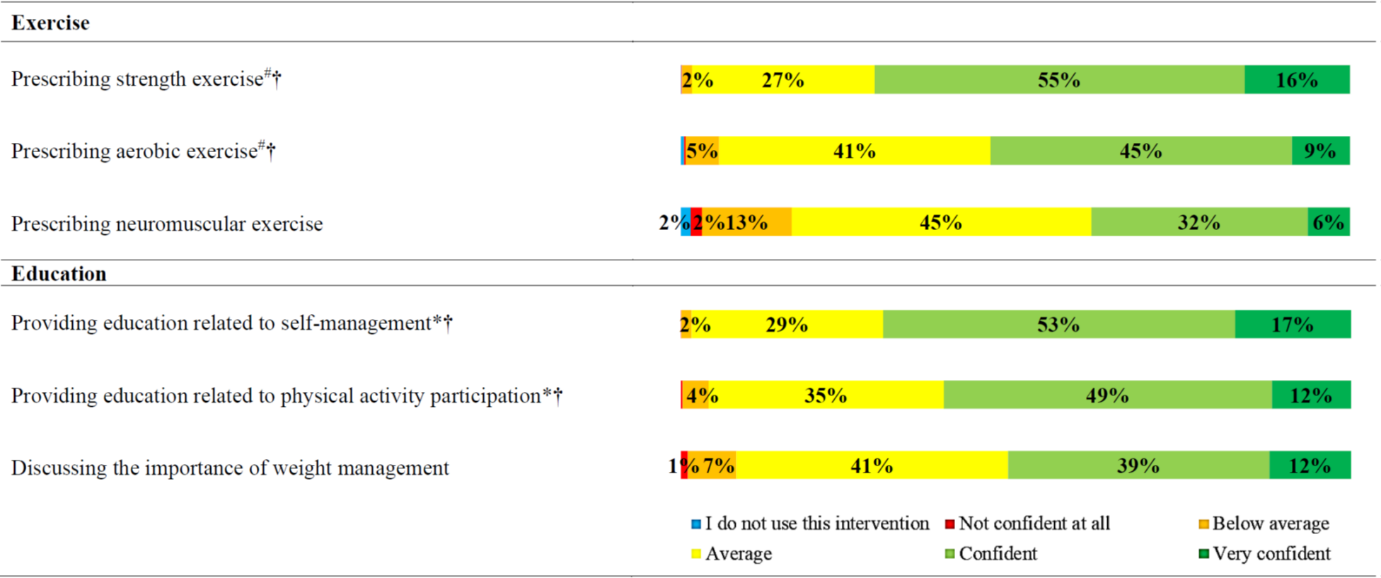
**

**Figure 3** Reported confidence to implement specific components of education and exercise therapy for people with knee osteoarthritis.


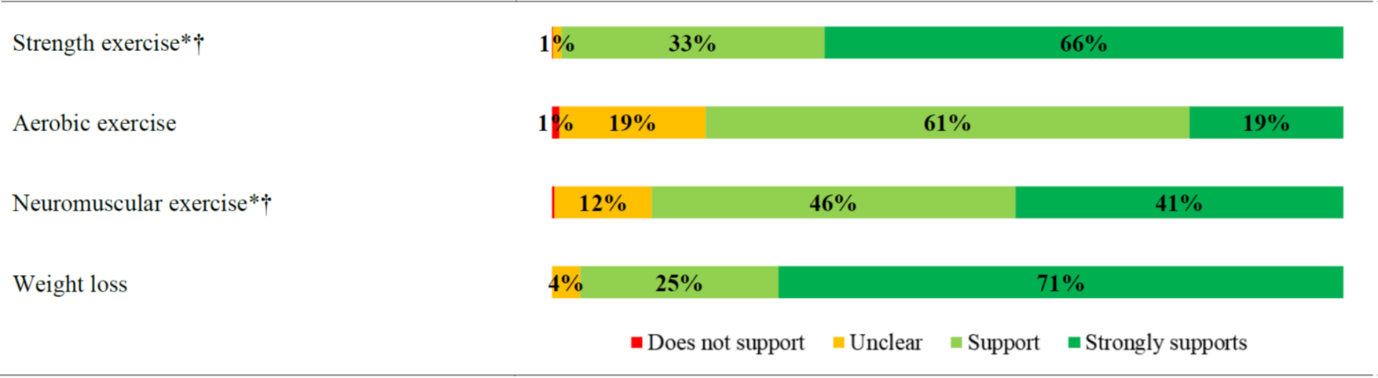


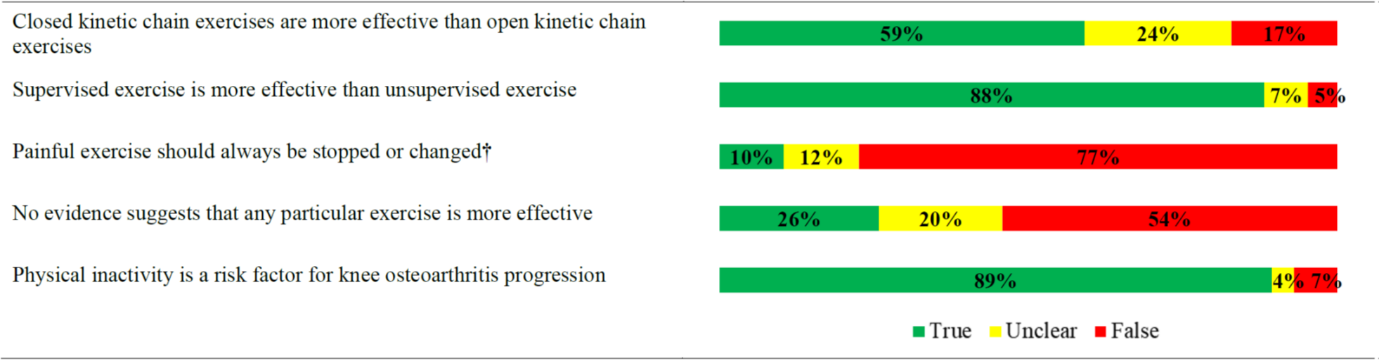


**Figure 4** Beliefs about evidence supporting specific exercise prescription and weight management; and exercise prescription principles and physical activity.
